# Tobacco consumption behavior change during the COVID-19 pandemic is associated with perceived COVID threat

**DOI:** 10.21203/rs.3.rs-3143401/v1

**Published:** 2023-07-06

**Authors:** Hollyann F. Loui, Joshua Li, Nicholas J. Jackson, Lauren E. Wisk, Russell G. Buhr

**Affiliations:** David Geffen School of Medicine at UCLA; David Geffen School of Medicine at UCLA; David Geffen School of Medicine at UCLA; David Geffen School of Medicine at UCLA; David Geffen School of Medicine at UCLA

**Keywords:** Tobacco, smoking, vaping, COVID-19

## Abstract

**Rationale:**

Tobacco use is a risk factor for COVID-19 adverse outcomes. Despite health implications, data conflict regarding COVID-19 and tobacco consumption. We present results from a survey of health behaviors during the pandemic to identify how COVID-19 influenced tobacco use.

**Methods:**

A national internet-based survey was deployed between May-September 2020. We analyzed participants who reported current or former smoking. We tabulated change in tobacco use, whether changes related to COVID-19, and measures of anxiety, depression, and novel perceived COVID-19 threat scale. We employed multinomial logistic regression to determine associations between these items and tobacco consumption.

**Results:**

We identified 500 respondents who reported ever smoking previously, 150 of which were currently smoking. Of 220 participants who reported any use of vapes, 110 were currently vaping. Increased perceived threat of COVID-19 was associated with both increased (aRR_increase_ 1.75, 95% CI [1.07–2.86], P = 0.03) and decreased (aRR_decrease_ 1.72 [1.04–2.85], P = 0.03) tobacco consumption relative to no change. There were no significant relationships found between perceived threat of COVID-19 and vaping behavior.

**Conclusions:**

As perceived COVID-19 threat increased, people were more likely to increase or decrease their smoking than stay the same, even after controlling for anxiety and depression, both of which can affect smoking in either direction. Further study into motivators of increasing or decreasing affected tobacco consumption, and how barriers to care from safer-at-home policies and changes in care delivery moderate change in tobacco use will aid planning tobacco reduction interventions during the ongoing and future respiratory viral pandemics.

## INTRODUCTION

Tobacco use and other diseases including cardiovascular disease and COPD are risk factors for severe COVID-19 infection and adverse outcomes [1, 2]. Despite these known health implications, data conflict regarding the impact of the COVID-19 pandemic on tobacco consumption globally, both for traditional and electronic cigarettes [3–7].

COVID-19 was a new, strong motivator to decrease tobacco use, and higher perceived risk of COVID-19 is associated with increased interest in tobacco cessation [4, 5]. Those who deliberately decreased tobacco consumption during the early COVID-19 pandemic cited concerns about contracting COVID-19 and becoming severely ill due to smoking’s link to lung damage, fear of spreading COVID-19 to others, or a desire to be healthier as their motivation to reduce or quit smoking [3, 4, 8]. Even those reporting low knowledge about smoking as a risk factor for COVID-19 disease severity were incentivized to quit or reduce consumption due to the social restrictions COVID-19 placed on tobacco use: safer-at-home policies limited smoking with friends, at parties, and when publicly socializing [6, 7].

Desire to reduce or quit tobacco use do not always match outcomes, with a US study demonstrating that almost half of participants reported interest in quitting but a third of respondents ended up increasing their consumption [4]. This finding matches reports of increased cigarette sales during the pandemic [9, 10]. While COVID-19 presented a strong case for improving overall health, it has also been a significant source of psychological distress, which may account for increasing tobacco use, at least in part due to the positive association between smoking and depression and anxiety [11]. In an Italian survey, higher perceived stress levels were associated with increased smoking, in addition to other factors reflective of worsening mental health including decreased sleep, decreased quality of life, increased depression, and increased anxiety [12, 13]. Likewise, other risk factors for tobacco use include unemployment, lower income, and alcohol consumption [12].

In this manuscript, we present the results from a nationally administered survey that included questions about health behavior changes during the COVID-19 pandemic. Identifying and understanding how COVID-19 has influenced tobacco use could be a powerful tool toward the development of effective cessation strategies. Reducing tobacco use in the general population would elevate public health and decrease the global disease burden of many illnesses including COVID-19.

## METHODS

### Survey Design

We drafted and deployed an internet-based survey to engage community opinions on how COVID-19 had affected participants’ ability to work, socialize, maintain their health, and seek medical care, as well as sociodemographic information to inform our analyses. Survey questions were deployed on REDCap version 10.6.14 (Vanderbilt University, Nashville, TN) [14, 15] initially. They were subsequently professionally translated (International Contact, Berkeley, CA) into Spanish, Korean, Mandarin, Vietnamese, and Tagalog and migrated to Qualtrics XM (Qualtrics, LLC, Provo, UT) for multilingual support, which was not available in REDCap.

### Recruitment of Participants

We recruited 1,971 participants who completed the survey. The study was disseminated through convenience and snowball sampling using social media platforms, including Facebook, Twitter, Instagram, and LinkedIn, as well as via direct recruitment through partner organizations. Detailed methodology on the design, deployment, and recruitment of participants has been previously published [16]. Though our recruitment strategy primarily focused on groups in California (71% of our sample), eligibility was not restricted by location and all adults (age ≥ 18) were eligible. Recruitment for this wave of the survey opened on May 8, 2020 and closed on September 30, 2020. This study was approved by the UCLA Institutional Review Board (20–000683) and registered with ClinicalTrials.gov (NCT04373135). Informed consent was obtained from all participants at enrollment before completing the first wave survey.

### Statistical Analysis

The survey included questions on self-reported change in tobacco use, whether changes related to COVID-19, and measures of anxiety, depression, and novel perceived COVID-19 threat items. Perceived threat of COVID-19 was assessed with the questions: “I am afraid of the coronavirus (COVID-19)”, “I purposefully try not to watch or read news on coronavirus”, “I spend a huge percentage of my time trying to find updates online or on TV about coronavirus”, “I am afraid of the coronavirus”, “I am not worried about the coronavirus”, “I have felt more lonely or isolated from other people because of coronavirus”, and “I am worried that I or people I love will get sick from the coronavirus”. Response options were on a scale of 1 to 7, with 1 being “Not true at all”, 4 being “Neutral”, and 7 being “Very true”. There was also an option to respond “I’d prefer not to answer” for each question [16, 17]. A PHQ-2 (Patient Health Questionnaire-2) score greater than 3 was used to define depression [18]. A GAD-2 (Generalized Anxiety Score-2) score of greater than 3 was used to define anxiety [19].

Demographics including age, gender, education, and work were excluded from our statistical models to conserve statistical power in the setting of the small sample size and lack of significant association in bivariate analyses but were included for description of the cohort. In the case of perceived COVID threat, the items in the novel scale were collapsed using standard factor analysis, such that a weighted scale score was tabulated from the individual questions and centered upon the mean, where each point on the scaled battery equals 1 standard deviation difference in weighted score compared to the sample mean [16].

The analytic sample was limited to the subset of participants who currently smoke or use an e-cigarette/vape, decrementing our eligible analytic sample from 1,971 respondents to 500 who reported current tobacco use and 220 who reported current vape use, further decremented in our models where complete case analyses were employed. Bivariate analysis examined change in each of smoked or vaporized tobacco use (increased, decreased, no change) with depression and anxiety scores (PHQ-2, GAD-2), the perceived COVID threat scale score, and baseline characteristics. Differences between continuous variables were evaluated with a Student’s t-test, and the differences between discrete variables were evaluated with a chi-squared test.

Multivariable multinomial logistic regression models using complete case analysis were used to examine how perceived COVID threat was associated with change in smoked or vaporized tobacco use after adjusting for other confounding variables (i.e. PHQ-2 or GAD-2 scores). A new composite binary variable measuring a high PHQ-2 or GAD-2 score (either score > 3) was created due to collinearity when PHQ-2 and GAD-2 scores were treated as separate variables. We compared an unadjusted model using only COVID threat to an adjusted model including age and mental health for our primary analyses. Because ~ 28% of respondents reporting current smoking had at least one missing datapoint on a covariate of interest, we utilized sensitivity analyses that incorporated an inverse probability weight (IPW) to account for factors associated with having missing data. This IPW was created using a logistic regression model for complete (1) vs missing (0) data based on these predictors: the presence of PHQ-2/GAD-2 scores, the participant’s change in mental health, and gender. All of the statistical analyses and modeling was done in R 4.2.1 with use of the nnet and ggplot2 packages [20, 21].

## RESULTS

### Cohort Demographics

Our sample included participants who reported any lifetime use of tobacco products (cigarettes or e-cigarettes/vapes), of whom 500 (88%) reported any cigarette use and 220 (39%) reported any e-cigarette/vape use. Of the individuals who reported cigarette use, 150 individuals had smoked cigarettes in the last 30 days, of which 23% smoked less, 33% smoked more, and 44% remained the same. Of the participants who reported current smoking (smoking in the last 30 days), 59% identified as female. The average age of those who reported no change to smoking was 46 years old (SD = 16), increased smoking was 41 years old (SD = 13), and decreased smoking was 50 years old (SD = 17).

We identified 220 participants who reported ever using vapes or e-cigarettes. 110 participants reported using vape or e-cigarettes in the last 30 days and were labelled as current users. Of these, 27% vaped less, 24% vaped more, and 49% remained the same. Forty-five percent of participants identified as female. The average age of participants who reported no change to vaping was 43 years old (SD = 15), increased vaping was 40 years old (SD = 13), and decreased vaping was 46 years old (SD = 16).

### Change in Tobacco Smoking Behavior

A majority (56%) of respondents changed their smoking behavior, and of those who changed 60% reported smoking more than prior while 40% smoked less. Respondents who decreased smoking were, on average, 4 years older than those that did not change smoking and 9 years older than those who smoked more (P = 0.04, Table 1). We did not observe statistically significant differences in smoking behavior across self-reported gender identity, race, ethnicity, education, or employment in our respondents (Table 1). Those who screened positive for depression by PHQ-2 score > 3 were almost twice (64% vs. 36%) as likely to increase smoking. Those who screened positive for anxiety by GAD-2 score > 3 were three times (76% vs. 24%) as likely to smoke more and slightly less (41% vs. 59%) inclined to smoke less than non-anxious counterparts in bivariate analyses (P = 0.03). Because there did not appear to be a significant relationship between smoking changes and demographics including gender, race/ethnicity, education, and work status in our bivariate analyses, these variables were not adjusted for in our final models.

**Figure 1:**
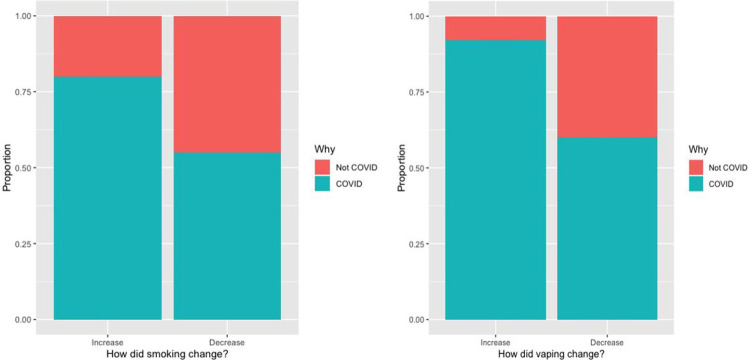
Self-reported change in smoking behavior and whether the change was influenced by COVID-19. Change in smoking behavior was reported by respondents as more likely to be due to COVID-19 than not (for both increased and decreased use).

In our unadjusted model, each standard deviation increase above the survey population mean perceived threat of COVID-19 measured by the novel COVID threat scale score was significantly associated with both increased (RR_increase_ 1.71, 95% CI [1.07–2.74]) and decreased (RR_decrease_ 1.77 [1.07–2.92]) tobacco consumption relative to no change suggesting a U-shaped relationship between perceived COVID threat and smoking behaviors (Table 2),. After controlling for the presence of either depression or anxiety using an aggregate variable that combined screening positive for anxiety or depression based on GAD-2 and PHQ-2, respectively, and age, we still found significant relationships between both increased (aRR_increase_ 1.75, 95% CI [1.07–2.86]) and decreased (aRR_decrease_ 1.72, 95% CI [1.04–2.85]) smoking behavior and perceived threat of COVID-19 (Table 2). When examining how smoking behavior changed, and when asked directly whether the change was influenced by COVID-19, participants self-reported that their change in smoking behavior was more likely to be due to COVID-19 than not for both increased and decreased use (Fig. 1).

### Change in Vaping Behavior

Slightly more participants (51%) changed their vape or e-cigarette use than remained the same, and among those that change, more respondents reported decreasing their use (53%) than increasing (47%). In our bivariate analyses, only anxiety symptoms were associated with change in vaping behavior, where those who reported increase in vaping having a 1.2 point higher score on the GAD-2 than those who decreased their vaping and 1 point higher than those who did not change the amount they vape. Interestingly, depression did not have a significant relationship with vaping change. We did not observe significant relationships between self-reported vaping changes and demographics including age, gender identity, race/ethnicity, education, and work status. In our regression models, when similarly observed no statistically significant relationship between perceived COVID threat and vaping behaviors in either unadjusted or adjusted analyses (Table 3).

### Sensitivity Analysis

We fit inverse probability weighted (IPW) models for missing data to determine the effects of missingness of responses. In these models, we observed a minimally augmented effect of COVID-19 perceived threat on smoking increase, with decrease in smoking falling just below statistical significance. There was no observed change in sign in any covariate for our smoking models. No significant changes in effect were noted in the vaping analyses in the IPW models (Supplemental Tables 1–2).

## DISCUSSION

Our results suggest that perceived threat from to COVID-19 is associated with change in tobacco use, observed as both increased and decreased smoking. This concern around COVID-19 influenced people to change their tobacco behaviors, even after accounting for depression and anxiety. Although we were not able to find statistically significant relationships between worsening mental health and vaping behaviors, a similar pattern was observed. Fear of COVID-19 provides a strong incentive for decreased tobacco consumption: smoking is linked to lung damage which is a risk factor for COVID-19 disease severity, contracting the illness comes with concern for potentially spreading it to others, the social pressure from others to quit or reduce smoking, and a general desire to be healthier in the face of a pandemic, among other reasons. Moreover, social restrictions from safer-at-home policies and fear of contracting the illness from others limited venues for smoking, and the financial burdens of the pandemic may have forced many individuals to quit the expensive habit out of necessity [3–8, 22]. Others may have unknowingly cut down on their tobacco use due to social restrictions from stay-at-home policies, and therefore never intended to quit.

Interestingly, our study found that perceived threat of COVID-19 influenced participants to change their smoking behaviors rather remain the same, with majority increasing consumption rather than decreasing. Many studies have demonstrated a clear positive association between smoking and mental illness, so it is not surprising that the psychological stress of COVID-19 and the resultant anxiety, depression, and overall worsening mental health would be associated with increased tobacco and vaping use [11]. Historically, stress-provoking events with national or global impact like the terrorist attack of September 11, 2001 have been linked to increased tobacco use, and the COVID-19 pandemic has had significant negative impact worldwide. In addition, the COVID-19 pandemic saw increased rates of unemployment, lower income, and alcohol consumption: all risk factors for increased tobacco usage in other studies [12, 13, 23]. As such, the complex social, biological, and psychological dynamics associated with the COVID-19 pandemic had myriad effects on respondents, including influencing their tobacco behaviors to change, whether in a positive or negative direction.

Stress is linked to increased difficulty quitting tobacco, and the concomitant stress of the COVID-19 pandemic and challenge of fighting an addiction (smoking and/or vaping) likely posed a huge barrier to successful cessation for many users of vape and tobacco products [3]. Varenicline, a medication used for tobacco cessation, was subject to a nationwide voluntary recall by Pfizer in July 2021. Though beyond the time horizon for this analysis, given this issue, we expect that ongoing changes related to tobacco behaviors may have been further compounded by this recall and potentially posed yet another hurdle to successful tobacco reduction and cessation during the ongoing pandemic [24].

Prior to the pandemic, tobacco industry research found that younger people who smoke of higher education and income were more likely to reduce or quit smoking [8]. Generally, younger people who smoke are more successful at reducing tobacco consumption and quitting than their older counterparts. The pandemic contributed to even higher quit rates amongst younger people who smoke who were no longer able smoke with their friends at school or at bars or clubs, reported that mask wearing made smoking inconvenient, or lived with parents unaware of their smoking habit and therefore were forced to quit during pandemic-related restrictions [6]. In contrast, in our study younger respondents were more likely to increase smoking and vaping while older respondents were more likely to decrease use (based off the average ages of those who reported no change, increased, and decreased smoking or vaping behaviors). This could be due to older participants viewing their personal risk from COVID-19 as being higher than younger counterparts, providing additional incentive to quit. Our results did not show a significant relationship between tobacco behaviors and work, education, or self-reported gender. These trends may have been present but were possibly limited by the statistical power of the study, potentially contributing to type 2 error.

### Limitations

This study recruited through community-based participation using social media methods. While perhaps less generalizable than national probability samples, this method of recruitment is still known to generate valuable insights and may be particularly useful during a pandemic when recruitment to studies is more challenging in general [16, 25]. The survey did not monitor changes in tobacco or vape use over multiple time points, precluding measurement of the impact of the pandemic on tobacco use as the pandemic progressed.

## CONCLUSION

Prior to our study, there was limited data on how the COVID-19 pandemic had influenced tobacco use, and the existing data appeared to conflict. Our study found that increasing perceived COVID-19 threat had a U-shaped relationship with smoking: people were more likely to increase or decrease their smoking than stay the same. We were unable to determine potential mediators or moderators that could explain the direction an individual may be influenced to change their consumption. Further study into how COVID-19 affected tobacco consumption, and how barriers to care from safer-at-home policies and changes in care delivery will aid planning tobacco reduction interventions during the ongoing pandemic.

## Supplementary Material

Supplement 1

## Figures and Tables

**Table 1:** Characteristics of respondents who reported smoking or vaping and change thereof during the pandemic

	Smoking (N = 150)				Vaping (N = 110)			
	No Change (n = 66)	Smoking More (n = 50)	Smoking Less (n = 34)	P-value	No Change (n = 54)	Vaping More (n = 26)	Vaping Less (n = 30)	P- value
Age in years, mean (SD)	46 (16)	41 (13)	50 (17)	0.04	43 (15)	40 (13)	46 (16)	0.39
Sex, n (%)								
*Female*	39 (59%)	27 (54%)	22 (65%)	0.62	27 (50%)	8 (31%)	15 (50%)	0.22
*Male*	27 (41%)	23 (46%)	12 (35%)		27 (50%)	18 (69%)	15 (50%)	
Race/Ethnicity, n (%)								
*Not White*	11 (18%)	8 (16%)	9 (29%)	0.32	14 (26%)	2 (8%)	7 (24%)	0.18
*White*	51 (82%)	42 (84%)	22 (71%)		40 (74%)	23 (92%)	22 (76%)	
Education Level, n (%)								
*Associate's*	16 (24%)	12 (24%)	12 (35%)	0.60	12 (22%)	3 (12%)	3 (10%)	0.26
*Bachelor's*	21 (32%)	20 (40%)	11 (32%)		22 (41%)	8 (31%)	15 (50%)	
*Graduate Level*	29 (44%)	18 (36%)	11 (32%)		20 (37%)	15 (58%)	12 (40%)	
Employment, n (%)								
*Working*	33 (50%)	34 (68%)	21 (62%)	0.14	39 (72%)	21 (81%)	21 (70%)	0.62
*Not Working*	33 (50%)	16 (32%)	13 (38%)		15 (28%)	5 (19%)	9 (30%)	
COVID Threat Scale Score, mean (SD)	−0.47 (1.08)	0.04 (0.88)	0.06 (0.84)	0.02	−0.2 (1.04)	−0.11 (0.74)	0.21 (0.59)	0.17
PHQ-2 Score, mean (SD)	2.1 (1.8)	3.5 (1.7)	2.0 (2.0)	< 0.01	2.2 (2.1)	2.8 (2.1)	2.0 (1.4)	0.38
Depression Diagnosis, n (%)								
*No*	37 (59%)	13 (36%)	22 (67%)	0.03	27 (60%)	11 (61%)	20 (69%)	0.72
*Yes*	26 (41%)	23 (64%)	11 (33%)		18 (40%)	7 (39%)	9 (31%)	
GAD-2 Score, mean (SD)	2.3 (1.9)	3.8 (1.7)	2.5 (2.1)	< 0.01	2.8 (2.2)	3.8 (1.7)	2.6 (1.6)	0.04
Anxiety Diagnosis, n (%)								
*No*	37 (57%)	11 (24%)	20 (59%)	< 0.01	25 (48%)	7 (28%)	16 (53%)	0.14
*Yes*	28 (43%)	34 (76%)	14 (41%)		27 (72%)	18 (52%)	14 (47%)	

N.B.: PHQ-2 = Patient Health Questionnaire, 2 items; GAD-2 = Generalized Anxiety Disorder Questionnaire, 2-items; COVID Threat Scale = factor weighted perceived threat to COVID-19 (novel scale) where each point change is 1 SD change in participant response compared to mean of sample

**Table 2:** Change in smoked tobacco use based on perceived threat of COVID

	Unadjusted (N = 118)		Adjusted (N = 116)	
Tobacco Change	**Relative Risk (95% CI)**	**P-value**	**Relative Risk (95% CI)**	**P-value**
COVID Threat Scale Score				
*More v No Change*	1.71 (1.07, 2.74)	0.03	1.75 (1.07, 2.86)	0.03
*Less v No Change*	1.77 (1.07, 2.92)	0.03	1.72 (1.04, 2.85)	0.03
Age				
*More v No Change*			1.00 (0.97, 1.03)	0.96
*Less v No Change*			1.00 (0.97, 1.03)	0.93
Anxiety or depression indicator				
*More v No Change*			3.21 (1.13, 9.12)	0.03
*Less v No Change*			0.54 (0.2, 1.47)	0.22

**Table 3:** Change in vaping behavior based on perceived threat of COVID and other variables

	Unadjusted (N = 88)		Adjusted (N = 87)	
Vaping Change	**Relative Risk (95% CI)**	**P-value**	**Relative Risk (95% CI)**	**P-value**
COVID Threat Scale Score				
*More v No Change*	1.11 (0.61,2.02)	0.73	1.03 (0.55, 1.95)	0.93
*Less v No Change*	1.87 (0.96, 3.67)	0.07	1.89 (0.97, 3.68)	0.06
Age				
*More v No Change*			0.99 (0.95, 1.03)	0.60
*Less v No Change*			1.00 (0.96, 1.03)	0.89
Anxiety or depression indicator				
*More v No Change*			1.34 (0.42, 4.28)	0.62
*Less v No Change*			0.72 (0.26, 2.02)	0.53

## What is already known on this topic

- Prior to this study, there was little data about how tobacco consumption had changed in the setting of the COVID-19 pandemic, and the data that did exist conflicted.

### What this study adds

- This study examined tobacco and vaping behavior changes during the COVID-19 pandemic.
- We found that perceived threat of COVID-19 had a significant impact on increasing tobacco use and almost significant impact on decreasing tobacco use even after controlling for anxiety and depression, making tobacco reduction interventions and strategies even more integral as the pandemic continues.

### How this study might affect research, practice, or policy

- Recognizing that worsening mental health and perceived threat of COVID-19 both have negative impacts on tobacco use is critical for effective tobacco cessation strategies for the ongoing and future pandemics.
