# Supplementary material for: Tobacco consumption behavior change during the COVID-19 pandemic is associated with perceived COVID threat
Source: Res Sq. 2023 Jul 6:rs.3.rs-3143401. Preprint. [Version 1] doi: 10.21203/rs.3.rs-3143401/v1 (PMC10350210; doi:10.21203/rs.3.rs-3143401/v1)
Supplement: Supplement 1 [file NIHPPrs3143401v1-supplement-1.pdf]

Supplemental Appendix Tables

Supplemental Table 1. Change in smoked tobacco use based on perceived threat of COVID with inverse probability weighting for complete responses

N = 113	Model 1		Model 2		Model 3		Model 4		Model 5	
Smoking Change	**Relative Risk (95% CI)**	**P-value**	**Relative Risk (95% CI)**	**P-value**	**Relative Risk (95% CI)**	**P-value**	**Relative Risk (95% CI)**	**P-value**	**Relative Risk (95% CI)**	**P-value**
COVID Threat Scale Score (per SD change above mean)										
More v No Change	1.97 (1.03, 3.75)	0.04	1.97 (1.03, 3.74)	0.04	1.78 (0.91, 3.47)	0.09	1.70 (0.89, 3.23)	0.108	1.97 (1.03, 3.74)	0.93
Less v No Change	1.78 (0.93, 3.40)	0.08	1.78 (0.93, 3.41)	0.08	1.62 (0.84, 3.1)	0.15	1.69 (0.88, 3.24)	0.114	1.78 (0.93, 3.41)	0.08
Age (per year change)										
More v No Change			1.00 (0.96, 1.04)	0.90	1.02 (0.97, 1.06)	0.46	1.01 (0.97, 1.06)	0.55	1.00 (0.96, 1.04)	0.90
Less v No Change			0.99 (0.95, 1.04)	0.73	1.01 (0.96, 1.05)	0.76	1.00 (0.96, 1.05)	0.98	0.99 (0.95, 1.04)	0.73
PHQ-2 Score (per point change)										
More v No Change					1.67 (0.98, 2.82)	0.057				
Less v No Change					1.25 (0.72, 2.18)	0.422				
GAD-2 Score (per point change)										
More v No Change							1.56 (0.93, 2.63)	0.09		
Less v No Change							1.32 (0.75, 2.34)	0.34		

N = 113	Model 1	Model 2	Model 3	Model 4	Model 5	
Anxiety or depression indicator						
More v No Change					2.75<br>(0.70, 10.79)	0.15
Less v No Change					0.56<br>(0.16, 2.00)	0.37

Supplemental Table 2: Change in vaping behavior based on perceived threat of COVID with inverse probability weighting for complete responses

N = 87	Model 1		Model 2		Model 3		Model 4		Model 5	
Vaping Change	**Relative Risk (95% CI)**	**P-value**	**Relative Risk (95% CI)**	**P-value**	**Relative Risk (95% CI)**	**P-value**	**Relative Risk (95% CI)**	**P-value**	**Relative Risk (95% CI)**	**P-value**
COVID Threat Scale Score (per SD change above mean)										
More v No Change	0.96 (0.38,2.41)	0.93	0.95 (0.38, 2.42)	0.92	0.84 (0.32, 2.16)	0.71	0.83 (0.30, 2.25)	0.71	0.95 (0.38, 2.42)	0.92
Less v No Change	1.67 (0.65, 4.29)	0.29	1.64 (0.64, 4.20)	0.30	1.61 (0.57, 4.50)	0.37	1.87 (0.69, 5.07)	0.22	1.64 (0.64, 4.20)	0.30
Age (per year change)										
More v No Change			1.00 (0.94, 1.05)	0.88	1.00 (0.94, 1.07)	0.91	1.00 (0.95, 1.06)	0.94	1.00 (0.94, 1.05)	0.88
Less v No Change			1.01 (0.96, 1.06)	0.64	0.99 (0.94, 1.05)	0.84	1.01 (0.96, 1.06)	0.77	1.01 (0.96, 1.06)	0.64
PHQ-2 Score (per point change)										
More v No Change					0.98 (0.54, 1.78)	0.95				
Less v No Change					0.82 (0.46, 1.44)	0.48				
GAD-2 Score (per point change)										
More v No Change							1.41 (0.57, 3.50)	0.46		

N = 87	Model 1		Model 2		Model 3		Model 4		Model 5	
Less v No Change							0.69<br>(0.32, 1.50)	0.35		
Anxiety or depression indicator										
More v No Change									1.65<br>(0.32, 8.38)	0.55
Less v No Change									1.01<br>(0.25, 4.13)	0.99
